# 3D Viscoplastic Finite Element Modeling of Dislocation Generation in a Large Size Si Ingot of the Directional Solidification Stage

**DOI:** 10.3390/ma12172783

**Published:** 2019-08-29

**Authors:** Maohua Lin, Xinjiang Wu, Xinqin Liao, Min Shi, Disheng Ou, Chi-Tay Tsai

**Affiliations:** 1 Department of Ocean and Mechanical Engineering, Florida Atlantic University, Boca Raton, FL 33431, USA; 2 School of Engineering, Fujian Jiangxia University, Fuzhou 350108, China; 3 School of Electrical and Electronic Engineering, Nanyang Technological University, 50 Nanyang Avenue, Singapore 639798, Singapore; 4 Department of Material Science and Engineering, Guangxi Technology University, Liuzhou 345006, China

**Keywords:** hassen-sumino model, CRSS model, dislocation density, a cooling process, directly solidification process

## Abstract

Growing very large size silicon ingots with low dislocation density is a critical issue for the photovoltaic industry to reduce the production cost of the high-efficiency solar cell for affordable green energy. The thermal stresses, which are produced as the result of the non-uniform temperature field, would generate dislocation in the ingot. This is a complicated thermal viscoplasticity process during the cooling process of crystal growth. A nonlinear three-dimensional transient formulation derived from the Hassen-Sumino model (HAS) was applied to predict the number of dislocation densities, which couples the macroscopic viscoplastic deformation with the microscopic dislocation dynamics. A typical cooling process during the growth of very large size (G5 size: 0.84 m × 0.84 m × 0.3 m) Si ingot is used as an example to validate the developed HAS model and the results are compared with those obtained from qualitatively critical resolved shear stress model (CRSS). The result demonstrates that this finite element model not only predicts a similar pattern of dislocation generation with the CRSS model but also anticipate the dislocation density quantity generated in the Si ingot. A modified cooling process is also employed to study the effect of the cooling process on the generation of the dislocation. It clearly shows that dislocation density is drastically decreased by modifying the cooling process. The results obtained from this model can provide valuable information for engineers to design a better cooling process for reducing the dislocation density produced in the Si ingot under the crystal growth process.

## 1. Introduction

Si crystal is the primary materials for solar cell for green energy applications [1]. Typically, high dislocation density generated in Si crystal will decrease the photoelectric conversion efficiency, reliability and lifetime of the solar cells [2,3]. To get a lower dislocation density Si ingot at a lower cost, the effects from crystal growth process on the dislocation generation have to be understood and the dislocation density produced in the crystal can be anticipated. However, the dislocation generation process of Si crystal is a complex thermal viscoplasticity process. Many works have been done to predict the generation of dislocation density in the crystals [4,5]. The first model to predict dislocation generation during crystal growth is called the critical resolved shear stress model (CRSS) proposed by Jordan [6,7], where the dislocation generation in the crystal is assumed to be correlated to the excessive resolved shear stress during the crystal growth process. This model can provide a qualitative prediction of dislocation density distribution cumulated in the ingot even though the actual quantity of dislocation density cumulated in the silicon ingot cannot be directly predicted. Another numerical model was first developed by Tsai [8] based on the Hassen-Sumino model (HAS) model [9]. HAS model couples the microscopic dislocation dynamics with the macroscopic viscoplastic deformation and it can directly predict the actual dislocation density generation during crystal growth. Since then, HAS model has been widely employed to calculate the dislocation density quantification of Si, GaAs and InP crystal from various crystal growth processes [10,11,12]. However, their works were based on the quasi-steady-state two-dimensional model. Furthermore, most crystals being investigated are axisymmetric crystal in nature [12,13,14]. Chen [15,16] has developed a three dimensional finite volume algorithm for the prediction of dislocation density generation in a non-asymmetric silicon ingot. However, the finite volume method is not flexible in modelling complex geometry and boundary conditions of crystal growth process [17]. Therefore, in our modelling of very large 0.84 m × 0.84 m × 0.3 m block ingots grown by directional solidification process, a three-dimensional transient finite element analysis (FEA) model is needed to predict the dislocation density in the non-axisymmetric crystal. One original and one modified cooling process [2,18] are employed to this developed transient model to predict the dislocation densities from the Si crystal. The results from the original cooling process will be compared with the results obtained from the CRSS model to demonstrate the validity of this developed model. It will be furthermore compared with the results from the modified cooling process to check out the initial cooling process. This developed finite element model is expected to provide a meaningful tool for engineers and scientists to design crystal growth and cooling processes for growing large size low dislocation density crystals.

## 2. Three Dimensional Viscoplastic Finite Element Model

The finite element model is developed from the Hassen-Sumino (HAS) model. In the Hassen-Sumino model [2,9,19,20,21], the viscoplastic strain components $\varepsilon_{ij}^{c}$, the viscoplastic rate component ${\dot{\varepsilon }}_{ij}^{pl}$, and dislocation density multiplication rate ${\dot{N}}_{m}$ are given as:

(1) $$\varepsilon_{ij}^{pl}=\overset{t}{\underset{0}{\int }}{\dot{\varepsilon }}_{ij}^{pl}dt$$

(2) $${\dot{\varepsilon }}_{ij}^{pl}=\frac{1}{2}bk_{0}N_{m}e^{\left(-Q/kT\right)}{\left(\sqrt{J_{2}}-A\sqrt{N_{m}}\right)}^{p}\frac{S_{ij}}{\sqrt{J_{2}}}$$

(3) $${\dot{N}}_{m}=Kk_{0}N_{m}e^{\left(-Q/kT\right)}{\left(\sqrt{J_{2}}-A\sqrt{N_{m}}\right)}^{p+\lambda }$$

(4) $$J_{2}=\frac{S_{ij}S_{ij}}{2},$$

where *k* is the Boltzmann constant that is 8.617 × 10^−5^ eV·K, *K*, *k*_0_ and λ are material constants of Si and given to be 3.1 × 10^−4^ *m/N*, 8.6 × 10^−4^ $m^{2p+1}N^{-p}s^{-1}$ and 1, respectively. *N_m_* is mobile dislocation density, *A* (strain hardening coefficient) is 4 *N·m^−1^*, *Q* (activation energy) is 2.2 eV, *p* (the stress exponent) 1.1, *b* (Burgers vector of Si) is 3.8 × 10^−10^ m, *S_ij_* is the deviatoric stress component, and $\sqrt{J_{2}}$ indicates the equivalent stress. ${\dot{N}}_{m}$ and ${\dot{\varepsilon }}_{ij}^{pl}$ is set to zero when $\sqrt{J_{2}}-A\sqrt{N_{m}}\leq 0$.

Since the dislocation multiplication during the cooling stage is a transient process in nature, a nonlinear three dimensional model is made to get dislocation densities in the ingot. The model can be formulated as [22]:

(5) $${\left[K\right]}_{n}\;{\left\{\Delta d\right\}}_{n}={\left\{\Delta F\right\}}_{n}$$

where

(6) $${\left[K\right]}_{n}=\int_{V}{\left[B\right]}^{T}\;{\left[\hat{D}\right]}_{n}\;\left[B\right]\;dV$$

and

(7) $${\left\{\Delta F\right\}}_{n}=\int_{V}{\left[B\right]}^{T}\;{\left[\hat{D}\right]}_{n}\;\left({\left\{{\dot{\varepsilon }}^{c}\right\}}_{n}\;\Delta t{\;}_{n}+{\left\{\Delta \varepsilon^{th}\right\}}_{n}\right)\;dV$$

In Equations (5)–(7), {Δ*d*}_n_ is the displacement, [*B*] is the matrix, {Δ*ε^th^*}_n_ is the thermal strain and {Δ*F*}_n_ is the equivalent load in a Δt_n_ = t_n+1_ − t_n_. The viscoplastic material matrix is given by:

(8) $${\left[\hat{D}\right]}_{n}={\left({\left[D\right]}^{-1}+\theta {\left[\frac{\partial {\dot{\varepsilon }}^{c}}{\partial \sigma }\right]}_{n}\Delta t_{n}\right)}^{-1}$$

[*D*] is the elastic material matrix. After calculating {Δ*d*}_n_, the displacement, residual stresses components and dislocation densities at time t_n+1_ can be calculated from:

(9) $${\left\{d\right\}}_{n+1}={\left\{d\right\}}_{n}+{\left\{\Delta d\right\}}_{n},$$

(10) $${\left\{\sigma \right\}}_{n+1}={\left\{\sigma \right\}}_{n}+{\left\{\Delta \sigma \right\}}_{n},$$

(11) $${\left(N_{}\right)}_{n+1}={\left(N_{}\right)}_{n}+{\left({\dot{N}}_{}\right)}_{n}\Delta t_{n}.$$

The initial dislocation density was assumed as 1.0 × 10^6^ m^−2^ in this paper.

## 3. Results and Discussions

**Figure 1:**
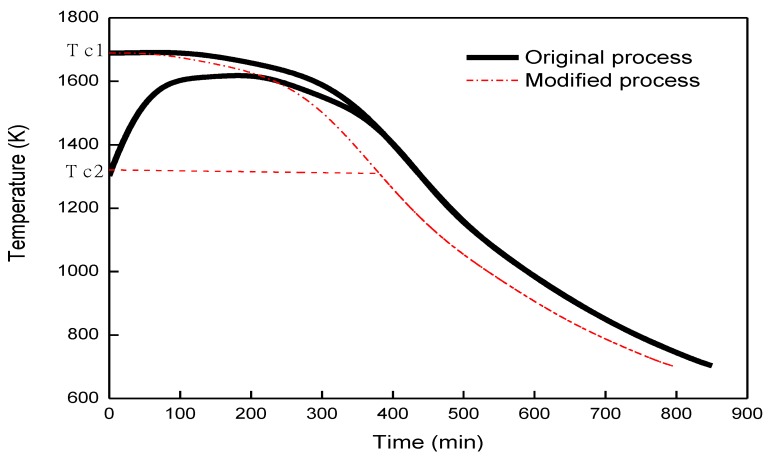
Temperature diagram (Tc1 and Tc2) with time under original and modified cooling process.

**Figure 2:**
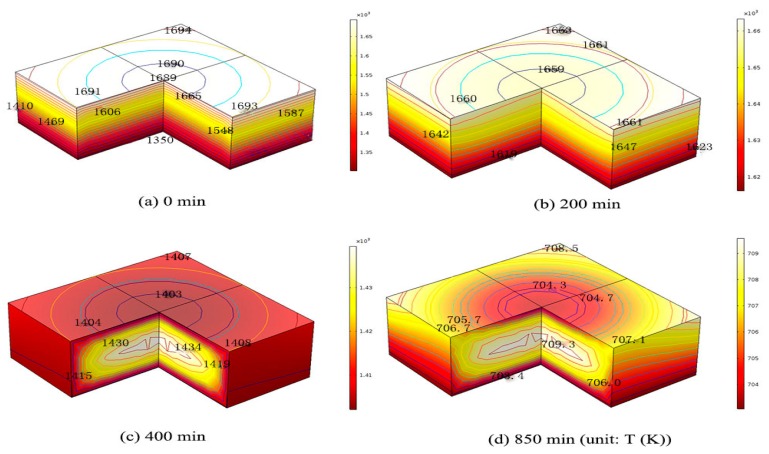
Temperature distribution of 0.84m × 0.84 m × 0.3 m silicon ingot at (**a**) beginning (0 min), (**b**) 200 min, (**c**) 400 min, and (**d**) end of the original cooling process (850 min).

One very large size Si ingot, 0.84 m × 0.84 m × 0.3 m is used to get the dislocation densities from the ingot during the cooling stage. One original and one modified cooling process during the growth of Si ingot are adopted to calculate the temperature field in the silicon crystal [2,18]. In the cooling process, the ingot top central temperature Tc1 and the bottom central temperature Tc2 are given in Figure 1 [2,18]. The temperature distribution in the silicon ingot is calculated by heat transfer module from commercial software COMSOL Multiphysics 5.2 (Burlinton, COMSOL) [18]. The temperature distribution in the silicon caused by the cooling stage at different time steps as shown in Figure 2 are then coupled with the developed three-dimensional FEA model to get the dislocation densities in the silicon.

**Figure 3:**
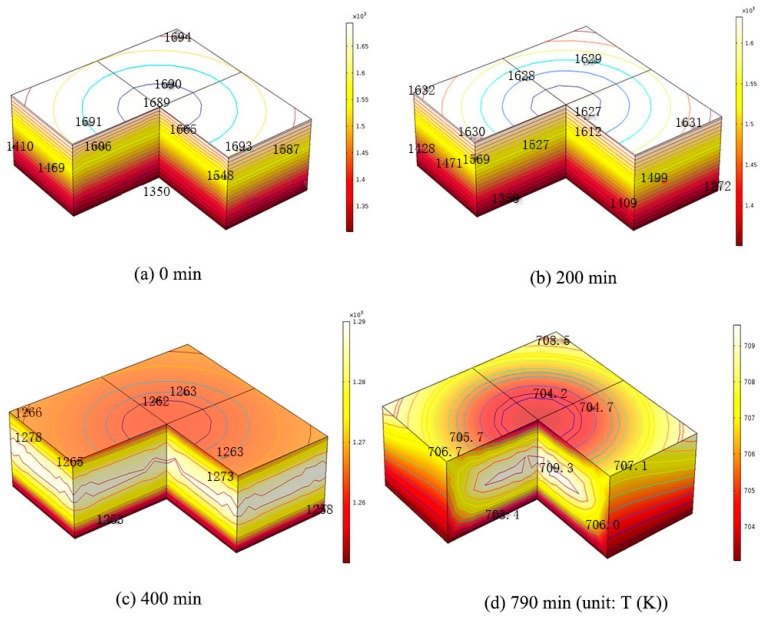
Temperature distribution of 0.84 m × 0.84 m × 0.3 m silicon ingot at (**a**) beginning (0 min), (**b**) 200 min, (**c**) 400 min, and (**d**) end of the modified cooling process (790 min).

The temperature distributions caused by the cooling stage are shown in Figure 2. Figure 2a shows the initial temperature distribution of 0.84 m × 0.84 m × 0.3 m silicon ingot at the beginning of the original cooling process. The temperature distribution shows a four-fold symmetry in the x-y plane. The value gradually increases from 1350 K at the center of the bottom surface of ingot to 1694 K at the four corners of the top ingot surface. The temperature in the original process is higher than 1073 K. The excessive stress will lead to the multiplication of dislocations in the ductile silicon crystal, and then the crystal deformation and stress release. After the 200th, 400th, and 850th min of cooling, the value of the maximum temperature difference between the bottom and top ingot decreases to about 40 K, 32 K, and 7 K, respectively as shown in Figure 2b–d. Figure 3a shows the initial temperature distribution of 0.84 m × 0.84 m × 0.3 m silicon ingot at the beginning of the modified cooling process, where the temperature distribution is the same with Figure 2a from the original cooling process. After 200th, 400th, and 790th min (the minute is not consistent with Figure 1 of cooling, the value of the maximum temperature difference between the bottom and top ingot decreases to about 230 K, 31 K, and 7 K, respectively as shown in Figure 3b–d). Compared with the original cooling process, the temperature decrease in the modified cooling process is much smooth during the first 200 min, which will cause less generation of dislocation density in the modified cooling process theoretically.

**Figure 4:**
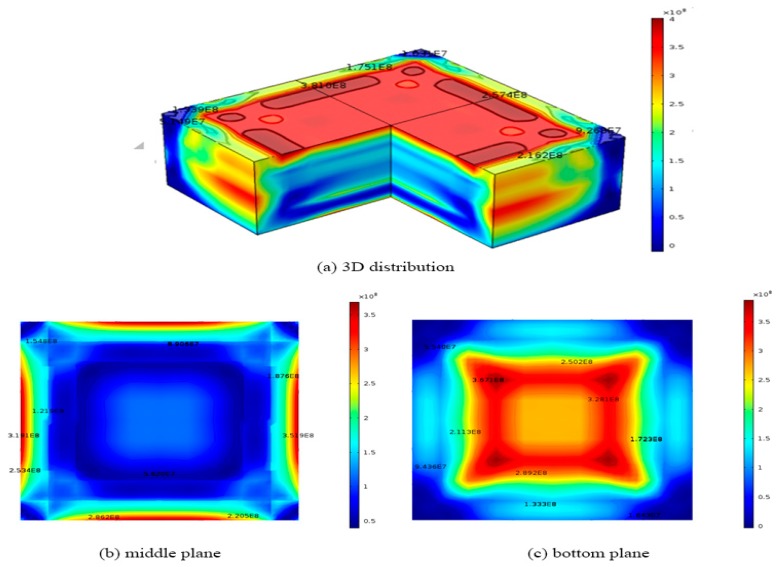
Dislocation density distributions of 0.84 m × 0.84 m × 0.3 m ingot at the end of the cooling process obtained from Hassen model with the original cooling process (**a**) 3D distribution; (**b**) middle plane; (**c**) bottom plane.

The temperature field caused by the cooling process at different time steps as shown in Figure 2 are then coupled with the developed three-dimensional FEA model to calculate the dislocation densities from in the silicon. Figure 4 shows the final dislocation density distribution in the 0.84 m × 0.84 m × 0.3 m silicon ingot calculated by Hassen model. On the top surface as shown in Figure 4a, the red region has a higher dislocation density of about 4.0 × 10^8^ m^−2^, while the lower dislocation density of about 9.3 × 10^7^ m^−2^ occurs near the edges of the top surface. Figure 4b shows final dislocation density variation on the middle surface (z = 0.15 m) of the ingot, where the blue region has a lower dislocation density of about 5.0 × 10^7^ m^−2^, while the largest dislocation density of about 3.5 × 10^8^ m^−2^ occurs at the edges of the middle surface as shown in Figure 4b,c shows the final dislocation density variation on the bottom plane (z = 0 m) of the ingot during the original cooling process, where the red region has a higher dislocation density of about 3.7 × 10^8^ m^−2^, while the lower dislocation density of about 4.7 × 10^7^ m^−2^ occurs near the edges of the bottom. The results are in good agreement with the reported experimental data [23].

**Figure 5:**
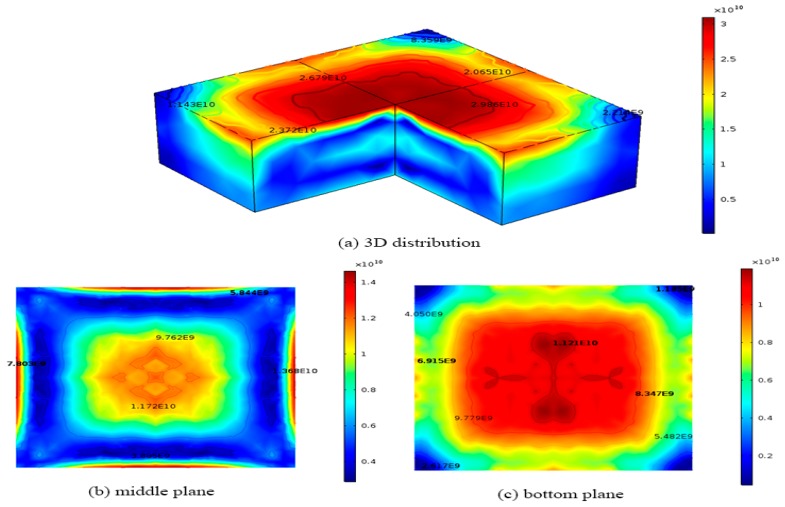
Dislocation density distributions of 0.84 m × 0.84 m × 0.3 m ingot at the end of the cooling process obtained from the critical resolved shear stress model (CRSS) model with the original cooling process (**a**) 3D distribution; (**b**) middle plane; (**c**) bottom plane.

We also compared the results obtained from our HAS model with those derived from the CRSS model [18]. In order to obtain a representative quantity of dislocation density, the CRSS model is derived by multiplying the excessive elastic stresses by a dislocation multiplication factor derived from HAS model [18]. Since the CRSS model does not consider the plastics deformation during the cooling process, the stress relaxation will not occur and the calculated dislocation density will be overestimated. However, the results from CRSS model can still predict the correct trend and qualitative distribution of dislocation density. The dislocation densities are solved by the CRSS model generated by the original cooling process in a 0.84 m × 0.84 m × 0.3 m silicon ingot. in Figure 5, the maximum dislocation density is about 3.1 × 10^10^ m^−2^ on the top surface of the silicon, while the minimum dislocation density is about 1.2 × 10^9^ m^−2^ on the bottom surface. On the top surface, the red region has a higher dislocation density of about 3.1 × 10^10^ m^−2^, while the lower dislocation density of about 1.1 × 10^10^ m^−2^ occurs near the edges of the top surface as shown in Figure 5a. Figure 5b shows final dislocation density variation on the middle surface (z = 0.15 m) of the ingot during the original cooling process, where the blue region has a lower dislocation density of about 3.8 × 10^9^ m^−2^, while the largest dislocation density of about 1.4 × 10^10^ m^−2^ occurs at the edges of the middle surface as shown in Figure 5b. Figure 5c shows the final dislocation density variation on the bottom plane (z = 0 m) of the ingot during the original cooling process, where the red region has a higher dislocation density of about 1.2 × 10^10^ m^−2^, while the lower dislocation density of about 1.2 × 10^9^ m^−2^ occurs near the edges of the bottom surface as shown in Figure 5c. The results show that the distribution pattern of dislocation density obtained from both models is similar except the magnitude of dislocation densities obtained from the CRSS model is 100 times higher than those obtained from this developed model. The dislocation density distribution pattern from both models also shows a famous four-fold symmetry. These results are expected since the CRSS model assumes the dislocation density generated by the crystal is proportional to the excessive elastic stress, which does not consider the stress relaxation due to plastic deformation caused by dislocation multiplication. Therefore, its dislocation density based on the accumulation of excessive elastic stress will be much higher.

**Figure 6:**
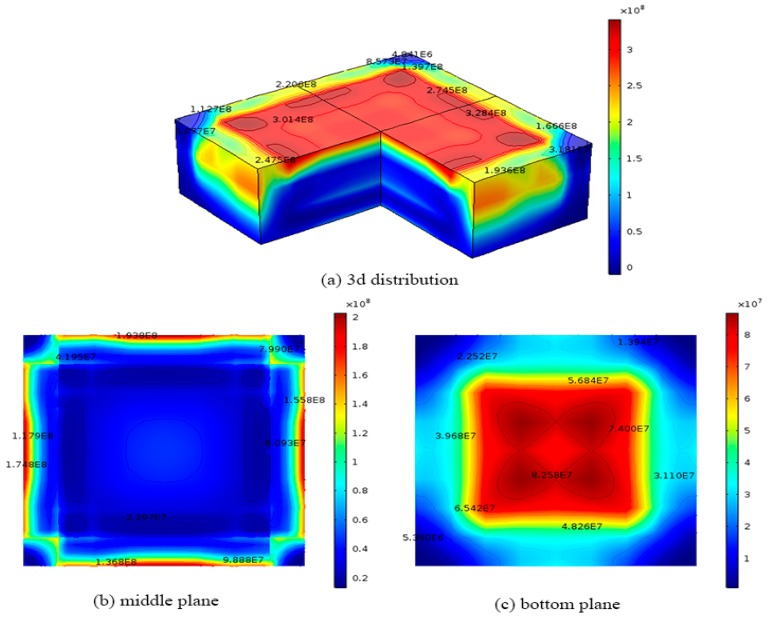
Dislocation density distributions of 0.84 m × 0.84 m × 0.3 m ingot at the end of the cooling process obtained from Hassen model with the modified cooling process (**a**) 3D distribution; (**b**) middle plane; (**c**) bottom plane.

Figure 6 shows the final dislocation density distribution in 0.84 m × 0.84 m × 0.3 m silicon obtained from the developed model with the modified cooling process. The maximum dislocation density of about 3.2 × 10^8^ m^−2^ is on the top surface of the silicon and the minimum dislocation density is about 1.4 × 10^7^ m^−2^ on the bottom surface of the silicon ingot. On the top surface, the red region has a higher dislocation density of about 3.2 × 10^8^ m^−2^ as shown in Figure 6a, which is 20% lower than that in the original cooling process, while the lower dislocation density of about 1.1 × 10^7^ m^−2^ occurs near the edges of the top surface. Figure 6b shows final dislocation density variation on the middle surface (z = 0.15 m) of the ingot during the original cooling process, where the blue region has a lower dislocation density of about 2.0 × 10^7^ m^−2^, while the largest dislocation density of about 2.0 × 10^8^ m^−2^ occurs at the edges of the middle surface as shown in Figure 6b, which is 42.9% lower than in the original cooling process. Figure 6c shows the final dislocation density variation on the bottom plane (z = 0 m) of the ingot during the original cooling process, where the red region has a higher dislocation density of about 8.3 × 10^7^ m^−2^, which is 77% lower than in the original cooling process, while the lower dislocation density of about 9.2 × 10^6^ m^−2^ occurs near the edges of the bottom surface as shown in Figure 6c.

**Figure 7:**
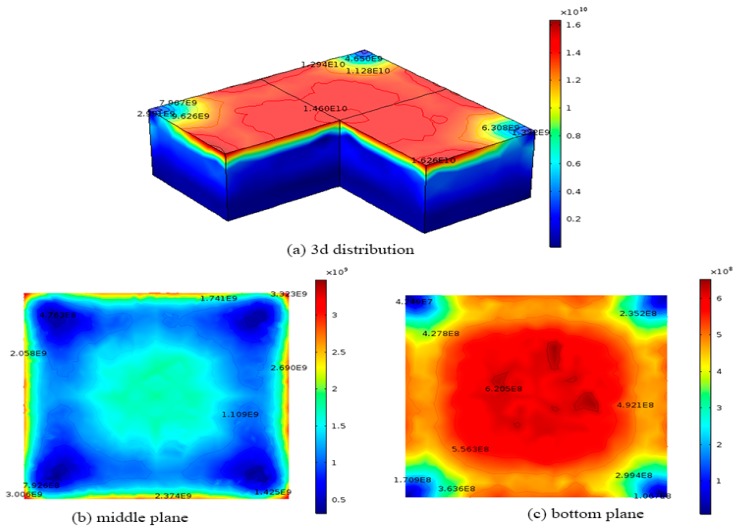
Dislocation density distributions of 0.84 m × 0.84 m × 0.3 m ingot at the end of the cooling process obtained from the CRSS model with the modified cooling process (**a**) 3D distribution; (**b**) middle plane; (**c**) bottom plane.

Figure 7 shows the final dislocation density distribution in the 0.84 × 0.84 × 0.3 m silicon ingot for CRSS model with modified cooling process. The maximum dislocation density is about 1.6 × 10^10^ m^−2^ on the top surface of the silicon, which is 46.7% lower than that in original cooling process. The minimum dislocation density is about 9.3 × 10^7^ m^−2^ on the bottom surface of the silicon ingot. On the top surface, the red region has a higher dislocation density of about 1.6 × 10^10^ m^−2^, while the lower dislocation density of about 1.1 × 10^9^ m^−2^ occurs near the edges of the top surface as shown in Figure 7a,b shows final dislocation density variation on the middle surface (z = 0.15 m) of the ingot during the original cooling process, where the blue region has a lower dislocation density of about 5.0 × 10^8^ m^−2^, while the largest dislocation density of about 3.3 × 10^9^ m^−2^ occurs at the edges of the middle surface as shown in Figure 7b, which is 76.4% lower than that in the original cooling process. Figure 7c shows the final dislocation density variation on the bottom plane (z = 0 m) of the ingot, where the red region has a higher dislocation density of about 6.1 × 10^8^ m^−2^, which is 95% lower than in the original cooling process, while the lower dislocation density of about 9.3 × 10^7^ m^−2^ on the bottom surface as shown in Figure 7c.

From Figure 4, Figure 5, Figure 6 and Figure 7, we can find that the distributions of dislocation density are almost similar as follows: Where the top and the bottom of the silicon hold the maximum dislocation density, while the four corners of the silicon hold the minimum dislocation density. However, the quantity in HAS model is much more accurate than that in the CRSS model. Also, the dislocation density and the scope of final dislocation density in CRSS model are quite higher than that in the HAS model as the stress does not release in CRSS model during the transient viscoplastic deform process. For the modified cooling process, the dislocation density is lower than that of the initial cooling stage both in the CRSS model and the HAS model. However, the HAS model can predict quantitatively and more accurate to be used in the cooling stage than the CRSS model.

## 4. Conclusions

A nonlinear three dimensional transient FEA model is successfully developed from the HAS constitutive model. The conventional CRSS model is used as a control group to compare with HAS model. The results obtained from both models show that the distribution of dislocation density is similar for both the original and modified cooling process. However, the magnitude of dislocation densities obtained from the HAS model is 100× lower than those obtained from the CRSS model. The maximum dislocation density obtained from the HAS model shows a reduction of 20% from original cooling process to modified cooling process, while the reduction is 46.7% from CRSS model. The reason that the HAS model predicts lower dislocation density and less dislocation reduction than the CRSS model is due to the fact that the CRSS model does not include the plastic deformations in its constitutive material model during numerical calculation. Therefore, the HAS model is a better model for predicting the quantity of dislocation generation during cooling process of crystal growth.
